# Conservative Treatment of the Antrum of Highmore

**Published:** 1903-12-15

**Authors:** Preston M. Hickey

**Affiliations:** Detroit, Mich.


### CONSERVATIVE TREATMENT OF THE ANTRUM OF HIGHMORE.

BY PRESTON M. HICKEY, M.D., DETROIT, MICH.

The patient contracted a severe cold with prominent nasal symptoms. In the course of the coryza the left maxillary sinus became infected with the usual symptoms. He was given a solution of adrenalin chloride, 1:4000, and directed to use it as a nasal spray every two hours. This was sufficient to keep the nasal passage free, and the mucous membrane of the maxillary outlet of the antrum sufficiently contracted to permit the proper drainage.

Resolution in this case was somewhat delayed, due to the fact that drainage during the day was interfered with by the erect position of the patient.

Acute inflammation of the antrum of Highmore, when due to nasal infection, should be treated conservatively for the first two weeks. The constringing effect of adrenalin chloride affords a valuable non-toxic means of reducing the swelling about the hiatus semilunaris.—*Therapeutic Gazette*, July, 1903.)

